# Building capacity in Clinical Epidemiology in Africa: experiences from Masters programmes

**DOI:** 10.1186/s12909-017-0885-4

**Published:** 2017-02-27

**Authors:** Taryn Young, Celeste Naude, Tania Brodovcky, Tonya Esterhuizen

**Affiliations:** 1 Centre for Evidence-based Health Care, Faculty of Medicine and Health Sciences, Stellenbosch University, Cape Town, South Africa; 2 Cochrane South Africa, South African Medical Research Council, Cape Town, South Africa; 3 Research Development and Support, Faculty of Medicine and Health Sciences, Stellenbosch University, Cape Town, South Africa

**Keywords:** Clinical Epidemiology, Capacity, Masters programmes, Africa

## Abstract

**Background:**

To describe and contrast programmatic offering of Clinical Epidemiology Masters programmes in Africa, to evaluate experiences of graduates and faculty, and assess if graduates are playing roles in research, practice and teaching of Clinical Epidemiology.

**Methods:**

We searched and identified relevant programmes, reviewed programmatic documentation, interviewed convenors and surveyed graduates. Participants provided informed consent, interviews with faculty were recorded and transcribed for analysis purposes, and graduates participated in an online survey.

**Results:**

Five structured Masters programmes requiring health science professionals to complete modules and research projects were assessed. Demand for programmes was high. Graduates enjoyed the variety of modules, preferred blended teaching, and regarded assessments as fair. Graduates felt that career paths were not obvious after graduating. Despite this, some have gone on to promote and teach evidence-based health care, and conduct and disseminate research. Areas of concern raised by faculty were quality assurance; research project initiation, implementation and supervisory capacity; staff availability; funding to support implementation and lack of experiential learning.

**Conclusion:**

Although faced with challenges, these programmes build capacity of health professionals to practice in an evidence-informed way, and conduct rigorous research, which are central to advancing the practice of Clinical Epidemiology in Africa.

**Electronic supplementary material:**

The online version of this article (doi:10.1186/s12909-017-0885-4) contains supplementary material, which is available to authorized users.

## Background

Clinical Epidemiology is the science of applying the best available research evidence to patient care [1]. It uses the methods of epidemiology to find scientifically valid answers to questions concerning diagnosis, prevention, therapy, prognosis and aetiology, thus improving the evidence base for the care of individual patients. Building capacity of health professionals to practice in an evidence-informed way [2, 3], and to conduct rigorous research, is central to advancing the practice and teaching of Clinical Epidemiology, with the ultimate aim of improving health outcomes and patient care.

Within the African region various continuing professional development initiatives focus on building capacity in evidence-informed practices [4] and work by organisations like Cochrane has been instrumental in raising awareness of evidence-based health care (EBHC) [5]. More advanced training at a Masters level has focussed on public health and epidemiology programmes. The majority of the latter programmes are concentrated in South Africa and Nigeria, support increased research productivity, and the number of these programmes have been shown as independent predictors of research productivity [6].

Little is known, however, about the extent and structuring of Masters level Clinical Epidemiology training within the African region and the experiences of both faculty and graduates. Furthermore, the roles that graduates of Clinical Epidemiology programmes are playing in both the practice and teaching of Clinical Epidemiology have not been assessed. This study’s objectives were to describe and contrast the programmatic offering of Masters programmes, offered at universities in Africa, with a core focus on Clinical Epidemiology, to evaluate the experiences of graduates and faculty, and to assess if graduates are playing roles in research, as well as the practice and teaching of Clinical Epidemiology.

## Methods

An internet search was conducted in Google using the terms ‘clinical epidemiology masters’ to identify Masters programmes with a core focus on Clinical Epidemiology that are being offered by universities in Africa. In addition, a research assistant looked at websites of all universities in the Southern, Western, Eastern, Northern and Central African region to identify all potential Masters programmes. The focus of the programmes had to be on Clinical Epidemiology – the science of applying the best available research evidence to patient care, which uses the methods of epidemiology to find scientifically valid answers to questions concerning diagnosis, prevention, therapy, prognosis and aetiology. General Masters in Public Health programmes that focus on the population level were not included unless they had a dedicated Clinical Epidemiology component.

The identified programmes’ convenors (also sometimes referred to as coordinators) were contacted by email to obtain programmatic documentation, for example, year book entries, programme outlines, and curriculum structures. From these, key information for pre-defined domains (Table 1) was extracted by one researcher and checked by another. The programmatic offerings were contrasted to show similarities and differences between the programmes.

**Table 1:** Data extracted from programmatic documentation

Name of Institution
Academic department/Division/School/Unit/Centre within which the programme is situated
Name of programme
Website
Programme coordinator and contact details
Year when programme started
Credit/equivalent value of the programme
Minimum duration of programme
Fulltime or part time
Admission
Admission requirements
Admission process
Annual student intake
Structured modules offered
Names of modules
Credit value of modules
Duration of modules
Objectives of modules
Type of offering: contact^a^/online/blended^b^
Assessment methods and frequency
Research projects
Types of research projects
Topics
Credit/equivalent weight
Overall number of students who successfully completed the programme
Total number of students currently in the programme

^a^Face: face

^b^Combination of contact and online

To supplement the information obtained from the review of programmatic documentation, interviews were conducted with programme convenors using a semi-structured interview guide (Table 2). These interviews were done by telephone or Skype. All interviews were conducted using the same interview guide, recorded with a digital voice recorder, with the consent of the interviewee, and transcribed for analysis purposes. Names of interviewees did not appear on the transcriptions. One researcher coded all transcripts, which were checked by another researcher. The researchers then carried out the data analysis and interpretation using thematic content analysis to identify key emerging themes. This iterative process of aggregation and interpretation were undertaken by the lead researcher and discussed with the rest of the research team.

**Table 2:** Semi-structured questionnaire for interviews with programme convenors

- For how long have you been involved in this Masters in Clinical Epidemiology programme?
- Briefly describe the programmatic offering
- Briefly describe the current methods of assessment
- How do you do quality assurance?
- How many graduates have you had for the programme?
- What problems and barriers do you encounter relating to the programme? How do you overcome these?
- What are your successes of the programme?
- Has this Clinical Epidemiology Masters programme influenced teaching and learning of evidence-based health care at undergraduate level?

Programme convenors were requested to provide the number of graduates from their respective programmes as well as their contact details. Graduates of the identified programmes were invited to participate in a short online survey (October 2014) (Additional file 1) that asked about their experiences of the programme, and the roles they have or are playing in research, practice and teaching of Clinical Epidemiology in Africa. The survey was emailed to graduates by either the research team, where email addresses were known, or by the convenors of the programmes. Quantitative data from the survey was analysed using descriptive statistics. Likert scale question scores were compared between the universities using non parametric Kruskal-Wallis tests and between the earlier and recent cohorts (2003–2011 and 2012–2014 respectively) using Wilcoxon rank sum tests. Data from open-ended questions were coded and summarised according to themes identified.

Ethics approval was obtained from Stellenbosch University Health Research Ethics Committee (N14/07/076) and institutional approval was obtained where necessary to contact graduates. Programme convenors and graduates were informed about the project and invited to participate. Participation by graduates in the online surveys was explicitly considered as informed consent and anonymity was ensured. For the qualitative interviews with faculty informed consent was obtained prior to the interview.

## Results

### Programmatic offering

We identified five eligible Clinical Epidemiology Masters programmes offered by the Universities of Cape Town, Pretoria and Stellenbosch in South Africa, University of Zimbabwe in Zimbabwe, and Makerere University in Uganda (Table 3). All of these were structured Masters programmes, offered mostly on a part-time basis, and requiring students to complete structured modules and a research project over a minimum period of 2 – 3 years.

**Table 3:** Programmatic description

Name of Institution	University of Cape Town	Makerere University	University of Pretoria	Stellenbosch University	University of Zimbabwe
Academic Department/Division/School/Centre where programme is situated	School of Public Health and Family Medicine	Department of Medicine	The School of Health Systems and Public Health	Community Health Division and Centre for Evidence-based Health Care	Department of Community Medicine
Name of programme	Master of Public Health (Clinical Research track)	Master of Science in Clinical Epidemiology and Biostatistics	Master of Science in Clinical Epidemiology	Master of Science in Clinical Epidemiology	Masters in Clinical Epidemiology
Year when programme started	MPH started in 1999. Clinical Epidemiology track started 2012 and incorporated into Epidemiology track 2014.	2000	2004	2008	1994
Credit/equivalent value of the programme	180 credits 1 credit = 10 hours	64 credit units (CU) 1 CU = 15 contact hours	180 credits 1 credit = 10 hours	180 credits 1 credit = 10 hours	180 credits 1 credit = 10 weighted hours
Fulltime or part-time	Fulltime or part-time	Fulltime	Part-time	Part-time	Part-time
Minimum duration of programme	1.5 to 2 years fulltime or 3 to 4 years part-time	2 years	2 years	2 years	3 years
Structured modules credit value	120 credits	55 CU	80 credits	120 credits	1400 weighted hours
Number of modules	Compulsory: 7 Elective: 3	Compulsory: 19	Fundamental: 3 Compulsory: 6 Elective: 3	Compulsory: 8 Elective: 2	Compulsory: 8
Type of offering	Contact (face:face) All modules are contact for a half week block (14–16 h) plus 8 or 9 sessions of 2 h spread throughout the semester	Contact (face:face) All modules are contact as lectures or classes	Contact (face:face) Contact lectures and individual computer-based tutorials and exercises, group work, assignments and self-study	Blended (face:face and online) Combination of contact lectures, e-learning using online learning platform, self-study (reading, formal and projects)	Blended (face:face and online) Distance learning with residential contact components twice per year (January and June)
Assessment	Formative assessments (50%) and summative assessments (50%)	Formative assessment (40%) and summative assessment (60%)	Formative assessments and summative assessments	Formative assessments (50%) and summative assessments (50%)	Formative assessment (25%) and summative assessment (75%) in year 1; only formative assessments in year 2
Research project credit value	60 credits	9 CU (4 CU for research proposal + 5 CU for dissertation)	100 credits	60 credits	400 weighted hours
Format	Research project resulting in 1 publishable peer reviewed paper	Dissertation	Research project resulting in 1 publishable peer reviewed paper	Research project resulting in 1 publishable peer reviewed paper	Dissertation
Number of graduates (as at October 2014)	4	104	31	40	10 – 12 per year

In two programmes the Master of Science (MSc) in Clinical Epidemiology programme was not a stand-alone programme and shared a number of modules with other Master’s degree programmes. For both Universities of Cape Town and Pretoria the Clinical Epidemiology programme was developed from the existing Masters in Public Health programmes, and specific modules on Principles of Clinical Epidemiology and Evidence-based Medicine were added to distinguish the Clinical Epidemiology programme from other programmes. The programmes at the other three universities were specifically developed as Clinical Epidemiology programmes.

Students typically completed a set of structured core (compulsory) modules and had a choice of 2 – 3 elective modules (Table 4). Comparing across programmes, modules on Epidemiology, Biostatistics, Research Methods, Economics and EBHC were common. Only one programme offered specific modules on Clinical Guidelines, Systematic Reviews and Meta-analysis, Randomised Controlled Trials and Diagnosis and Screening. Modules were offered using either contact (face:face) sessions with self-study, or blended (a combination of contact and online) sessions with self-study. Assignments and evaluations included both formative and summative assessments. Only one programme had a dedicated practical placement period where students got placed in various settings during which they need to develop the topic of their dissertation. Research Projects, which form between 22 and 55% of the total credit value of the five programmes, could be either primary studies or systematic reviews. Each student was linked with a specific project supervisor who provided guidance and supervision for the duration of the project. Three of the 5 programmes required students to submit their projects as publishable manuscripts while the other 2 programmes required full dissertations.

**Table 4:**
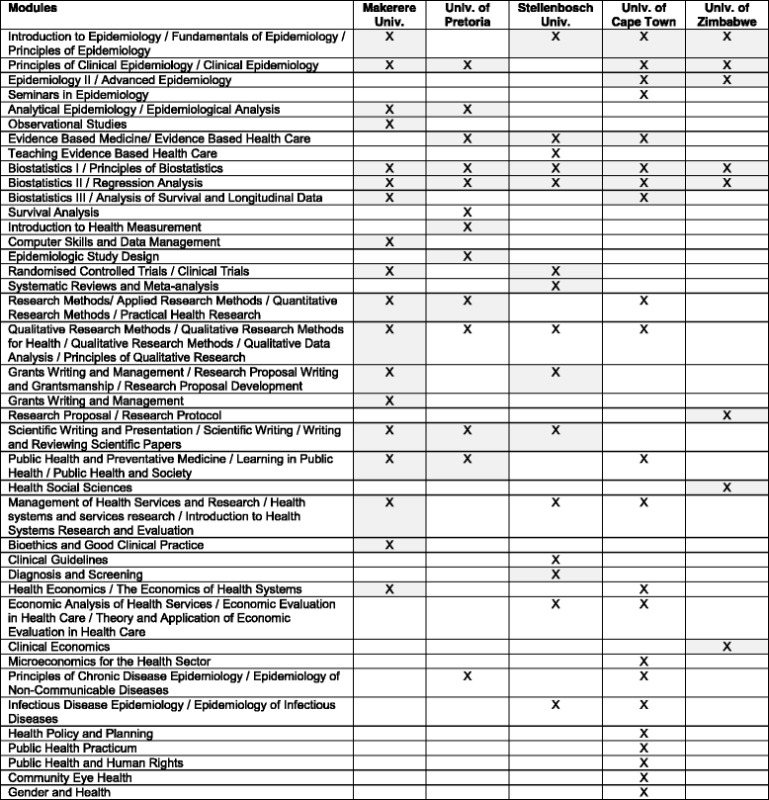
Matrix of structured modules offered by the five identified Masters in Clinical Epidemiology programmes

Note: Core (compulsory) modules are shaded in grey

### Demand for programme

Demand for the programmes was high, with most programmes having far more applicants than they could accept, as ascertained from interviews with programme convenors. The limitation was capacity and related to the lack of convenors, lecturers, and importantly, supervisors of research projects – thus limiting the number of students that could be enrolled.

*‘..It’s been enormous; we’ve had to squeeze people in. You know in Biostatistics I think there was about forty-five students and it’s difficult to maintain personalised teaching and being able to come around to everybody and answer everybody’s question and find a research topic for everybody and a supervisor for everybody…[convenor]’*

### Experiences of graduates and faculty

We were able to invite 183 graduates from four of the five programmes to participate in the online survey and 18% (33/183) responded (Table 5). They had various health science degrees including medicine, nursing, nutrition, optometry, emergency medical care as well as various medical specialities (for example paediatrics, psychiatry, surgery). Graduates enjoyed the academic and geographic diversity of fellow students and referred to peer interaction and a community of students that provided support to one another. They liked the range and variety of modules and found the modular block structure manageable and regarded assessments as fair (Table 6). Programmes that presented blended programmatic offerings, i.e. combining contact teaching with online sessions and self-study enabled those working in various countries/regions to enrol in the programme and also to participate actively in the modules while still continuing in their jobs. During contact sessions they especially liked the practical sessions, discussions and engagement with lecturers. There were no significant differences in student experiences of the programmes between the different universities.

**Table 5:** Survey responders

Variable		
Female; Male	14 female (42%); 19 male (58%)	
Age (mean)	36 years	
Previous qualifications	Emergency medical care	1
	Medicine	17
	Nursing	2
	Nutrition	4
	Optometry	1
	Pharmacy	2
	Sports Science	1
	Statistics, epidemiology	5
When graduated	2003	1
	2004	1
	2007	1
	2010	5
	2011	4
	2012	11
	2013	5
	2014	5
University	Makerere University	8
	Stellenbosch University	20
	University of Cape Town	3
	University of Pretoria	2
Time to complete the programme	2 years	16
	3 years	15
	4 years	1
	5 years	1

**Table 6:** Graduates’ experiences with Masters in Clinical Epidemiology programme (*n* = 33)

N (%)	Strongly disagree	Disagree	Neutral	Agree	Strongly agree
Application procedure					
The application procedures were clear			3 (9.0)	15 (45.5)	15 (45.5)
The application process was easy to follow			3 (9.0)	15 (45.5)	15 (45.5)
Structured modules					
There was a variety of modules			1 (3.0)	14 (42.4)	18 (54.6)
Duration of the modules was adequate		2 (6.1)	1 (3.0)	19 (57.6)	11 (33.3)
Assessments were fair		1 (3.0)	3 (9.1)	16 (48.5)	13 (39.4)
Research project					
Guidelines were clear			4 (12.1)	13 (39.4)	16 (48.5)
Support from supervisor was adequate	1 (3.0)	2 (6.1)	1 (3.0)	10 (30.3)	19 (57.6)
General					
Doing the MSc in Clinical Epidemiology influenced my career path		1 (3.0)	4 (12.1)	9 (27.3)	19 (57.6)

Even though most of the programmes are offered part-time, some students felt that there were too many contact sessions and some felt disadvantaged as they needed to use personal leave to attend these sessions and spend much funds to travel. They commended the quality of the lectures and viewed the lecturers, both local and international, as experienced, interested and willing to teach. Lecturers with experience in the field of Clinical Epidemiology were especially valued. Research project guidelines were clear and students highlighted that having a supervisor who offered dedicated guidance and support for the research project was advantageous. Graduates felt that career paths were not always obvious after completion of these programmes, specifically whether to branch into research, teaching or use it as an adjunct in clinical practice.

*‘Attending classes as a group allowed peer interaction and additional learning opportunities.’ [graduate]*

*‘It was sometimes challenging to attend all the sessions due to work commitments’ [graduate]*

*‘The contact sessions were adequate. The online learning platforms worked very well with the ability to access lectures from anywhere with the aid of the internet.’ [graduate]*

*‘Diversity of modules and diversity of the teaching staff coming from all over the world; this was amazing! Being in class with colleagues from diversity of background, who could understand me without judgment …was a key to my integration and my success. Finally and most importantly, being granted a scholarship the second year on my study was actually something I will always be grateful for, because it allowed me to have a clear mind, not worrying about fees to pay and only focus to my studies.’ [graduate]*

Key issues and areas of concern raised by faculty related to quality assurance, research project initiation and supervision, staff availability, funding to support programme implementation and lack of experiential learning. Quality assurance of the programmatic offering in two of the five programmes was done with external examiners who assessed the content of the modules, assignments and examinations prior to and subsequent to assessments. Student feedback was also sought and international benchmarking had been done by a few programmes. Other programmes, however, lacked in this area with some convenors saying that their programmes had never had quality assurance processes in place, and some saying that they used to have it but it had since fallen away due to overburdened staff.

*‘After 2006 quality assurance was more considered a luxury with the decrease of manpower and finding the most suitable people to apply quality assurance, but this is now being relooked at, with possibly a new approach.’ [convenor]*

The research project component of most of the courses was regarded as a challenge, and perceived as the largest barrier to successful and timely programme completion. Convenors raised concerns about competing priorities and their capacity to supervise many students, as well as to recruit additional suitable supervisors. The process of identifying relevant research topics was also raised as an issue as students tend to come up with a wide range of research projects, often without focus, and required a lot of support to refine their topics and projects. Furthermore, the ongoing management and coordination of many active and ongoing research projects and ensuring successful completion of projects by students was time consuming for supervisors.

*‘…coming up with the topic seems to be the hardest thing for them but we guide them in this process…’ [convenor]*

*‘…some of them go for five years on a Master’s programme you know, so we try and encourage them to complete it in three at most but many, most would I say, take longer than three years..… So that means also, there’s a huge group in the middle with clogging the system so to speak… knowing you taking in new people because you’re still busy supervising, three, four or five others that are still in the system for a number of years now.’ [convenor]*

*‘Process to identify a supervisor - this was a nightmare. The school did not suggest a supervisor, but left it to the students to approach and identify a supervisor. Therefore I had to 'beg' profs and lecturers to consider me, to which all of them (and I approached everyone in the school applicable to my MSc) said no, they were too busy or already had too many students. This was extremely disheartening and delayed my thesis work by two years!’ [graduate]*

In addition, both faculty and graduates identified the lack of opportunities for students in experiential learning – opportunities that would provide an ideal platform for students to apply their newly gained knowledge and to add depth of understanding of Clinical Epidemiology in practice.

*‘The students come and then they go but there’s no onsite exposure, onsite training, fieldwork kind of a thing in the training programme, so in a certain sense very theoretical in spite of everything that we try and do.’ [convenor]*

*‘It would be good to include field visits/placements, even if short, with some group projects, and many practical data analytical practices.’ [graduate]*

### Role graduates are playing in research, practice and teaching of Clinical Epidemiology in the African region

A large number of Clinical Epidemiologists had graduated. The majority of graduates who responded to the survey indicated that completing the MSc in Clinical Epidemiology positively influenced their career paths and 13 (39%) changed jobs after completing the degree. We compared the two year cohorts (2003–2011 and 2012–2014) using Wilcoxon rank sum tests. Between the year cohorts the item on “Doing Clinical Epidemiology influenced my career path” was significantly different. Cross tabulation showed that the significance lies in the 2003–2011 cohort being more likely to agree more strongly than the other cohort.

*‘I wanted to put to practice my newly acquired skills.’ [graduate]*

**Fig. 1:**
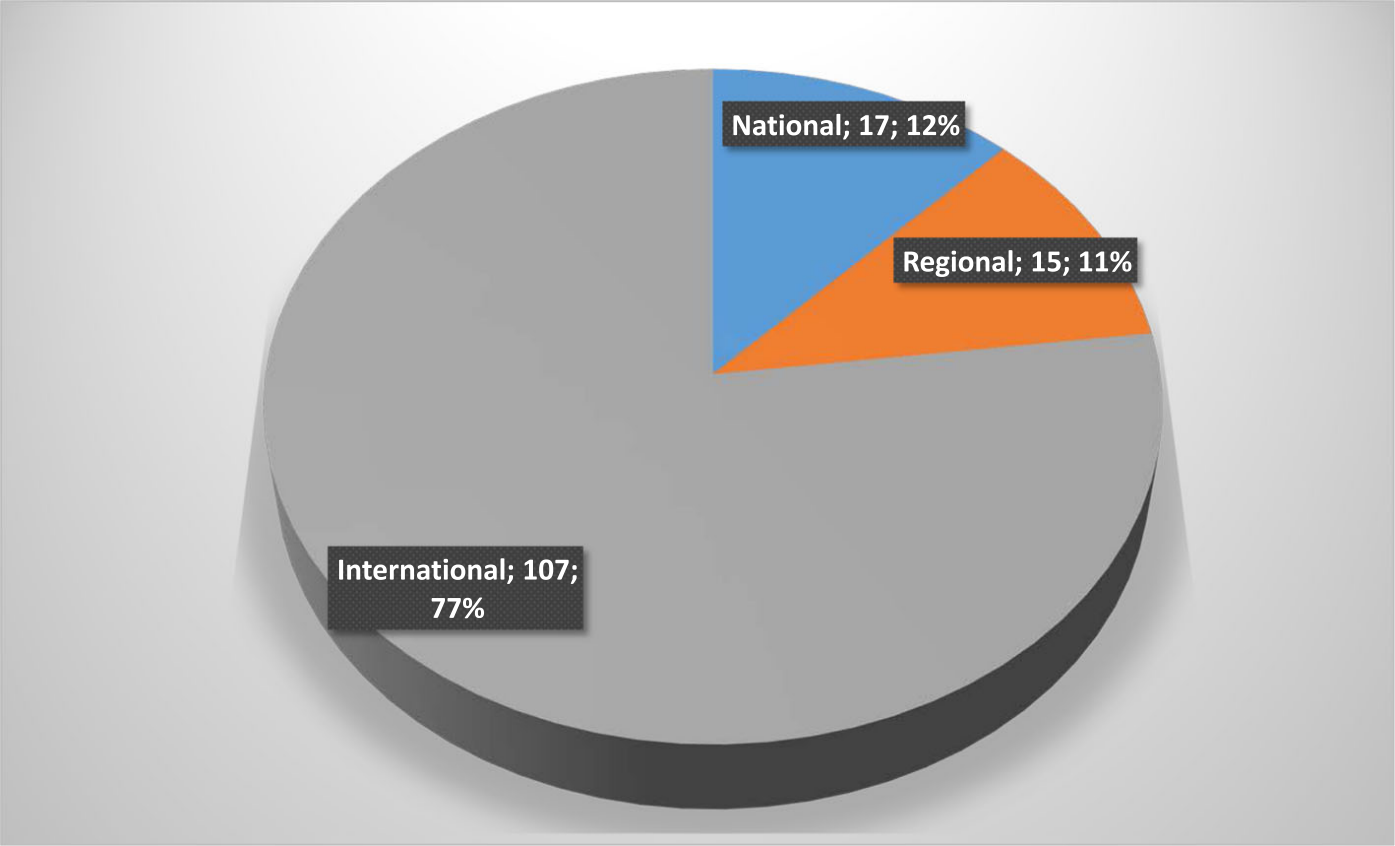
Total number of publications in national, regional and international journals of Masters in Clinical Epidemiology graduates (*n* = 28)

Graduates had gone on to practice EBHC in their positions of employment, and many occupy high level positions in governments and large companies and organisations, regionally and internationally, while others are embarking on Ph.D. studies. Some graduates were advancing research, with 85% of graduates who responded to the survey (28/33) being actively involved in the conduct and dissemination of research. They were conducting collaborative research on various issues related to the burden of disease in the African region and using a variety of research methodologies such as systematic reviews, surveys and diagnostic test accuracy studies. These graduates were presenting their findings at regional and international conferences and had collectively published more than 100 papers in various national, regional and international journals (Fig. 1).

*‘My mindset was changed as a practitioner to be able to use evidence based medicine through enthusiasm to search for information, understand results and their implication, critically appraise articles and know what applies to my patient setting.’ [graduate]*

Half of survey respondents were involved in teaching Clinical Epidemiology to both undergraduate and postgraduate students and they used various teaching and learning approaches including lectures, small group discussions, online learning and interactive learning strategies. Many of the graduates that responded were also involved in promoting the use of evidence in decision making through working with guideline developers, clinicians, policymakers and enhancing capacity to use evidence.

Faculty also perceived some level of indirect transfer of EBHC from the Masters programmes to teaching and learning in undergraduate programmes. This happened mostly via diffusion due to common lecturers at undergraduate and postgraduate levels, and also due to some programme graduates going on to become academic staff that could influence undergraduate programmes.

*“…the success stories is our resilience, so people training irrespective of all the economic difficulties that we have. Each year we have continued to train in not less than ten people. We have benefited in a local sense in an international sense and a lot of them are actually contributing to real science as it were.” [convenor]*

## Discussion

EBHC is of utmost importance in resource-limited settings [7]. It is widely recognised, not without criticisms [8], that all healthcare professionals need to be competent to practice in an evidence-informed way [2]. Masters level training in Clinical Epidemiology builds capacity in both using and conducting research to inform healthcare practices. In this study we described and contrasted the programmatic offering of five Masters in Clinical Epidemiology programmes offered at universities in Africa, and evaluated the experiences of graduates (Table 7) and faculty. Graduates are playing key roles in advancing research, teaching of EBHC and evidence-informed practices. Clinical Epidemiology programme structures align well with similar programmes in North America [9] and Australia [10] and the challenges experienced in African programmes also resonate with those of other programmes [9]. Key programmatic issues which came up were lack of experiential learning, limited funding to support programme implementation, limited bursaries and funding for students to cover fees and research projects, limited supervisory capacity for research projects (limited both in number of available and suitable supervisors and quality of supervision), lack of alignment of research project scope with expertise of supervisors, quality assurance and career pathing.

**Table 7:** Key messages

What is already known about this topic?
• Building capacity in Clinical Epidemiology supports evidence-informed practices
• Various Masters in Clinical Epidemiology programmes exist worldwide but experiences of these have not been formally evaluated
What we know after this paper?
• To the best of our knowledge this is the first assessment of Masters of Clinical Epidemiology programmes in the African region
• There is a high demand for Clinical Epidemiology Masters programmes
• Graduates are active in research, teaching and promoting evidence-informed practices
• Programme convenors highlighted the need for experiential learning and career pathing and that there is limited capacity to supervise research projects
• Collaborative efforts among academic programmes can draw on collective experience to augment development and delivery

Research outputs from Africa are increasing [6, 11] and graduates from Masters in Clinical Epidemiology programmes are contributing to this. Clinical Epidemiologists also play a key role [12, 13] in the many efforts to build EBHC capacity in low and middle income countries [14]. To this end it is important to have clear guidance on the scope of practice for Clinical Epidemiologists. Existing programmes and graduates can contribute to developing this scope of practice, however, from our study, there seems to be limited collaboration and engagement between programmes. Collaborative efforts among academic programmes can also draw on collective experience to augment development and delivery, especially in experiential learning and in building distance-learning facets, as requested by students, and evaluate its potential impact on the quality of graduates delivered.

There are still some unanswered questions. Is there a role for a community of practice for graduates after completing the programme? In terms of international benchmarking and standardising Clinical Epidemiology training programmes, what are the minimum core standards and content for Masters in Clinical Epidemiology programmes? Learning outcomes are defined for EBHC learning [15] however what are core learning outcomes for Clinical Epidemiology Masters programmes and is this adequate to equip a Clinical Epidemiologist?

To the best of our knowledge this is one of few studies that have been done to assess Master’s level training in Clinical Epidemiology. We searched widely to identify Clinical Epidemiology programmes. Our graduate survey, however, despite regular reminders and a lengthy time provided for response, was limited by a very low response rate and therefore may not adequately reflect the perceptions, experiences and current activities of graduates. Interviews were conducted with programme convenors and not with other faculty supporting the programmatic offering. Interviewing more faculty could enhance the depth of data. We have also not assessed the cost of programmes, the appropriateness of learning methods and environments, whether programmes are achieving their defined learning outcomes, financial models of the different programmes and the views of external stakeholders of the value of this type of capacity development programmes. Despite these limitations this research assessed existing Clinical Epidemiology Masters programmes using comprehensive assessment of programmatic documentation, interviews with convenors and a graduate survey, and provided information on the extent and structure of Masters level Clinical Epidemiology training within the African region as well as the experiences of both convenors and graduates.

## Conclusions

Although faced with some challenges, graduates enjoyed the programmatic offering and are playing key roles in advancing evidence informed practices in the African region.

## Additional file

Additional file 1: Graduate survey. The survey used to collect data from graduates (DOCX 15 kb)
