# Supplementary material for: Building capacity in Clinical Epidemiology in Africa: experiences from Masters programmes
Source: BMC Med Educ. 2017 Feb 27;17:46. doi: 10.1186/s12909-017-0885-4 (PMC5327556; doi:10.1186/s12909-017-0885-4)
Supplement: Additional file 1: — Graduate survey. The survey used to collect data from graduates (DOCX 15 kb) [file 12909_2017_885_MOESM1_ESM.docx]

Supplementary file: Graduate survey

***Online questionnaire (the questionnaire will be set up online)***

1. Age: ##
2. Gender: Male/Female
3. Qualifications: ______________________
4. At which University did you complete the masters in clinical epidemiology? __________________
5. When did you graduate (year)? ####
6. How long did it take you to complete the programme? ## years
7. Tell us about your current job:
   1. Where are you working? ____________________________________
   2. Describe your role: ______________________________________________
   3. How long have you been in this post? ## months ## years
   4. Have you changed jobs after you obtained your masters in clinical epidemiology? Yes / No
      1. If yes, why? ________________________________________
8. Experience with the masters in clinical epidemiology
   1. Indicate your response to the following statements related to your experience in doing the masters programme in clinical epidemiology by ticking the most appropriate option:

|  |  |  |  |  |  |
| --- | --- | --- | --- | --- | --- |
|  | **Strongly disagree** | **Disagree** | **Neutral** | **Agree** | **Strongly agree** |
| **Application procedure** |  |  |  |  |  |
| The application procedures were clear |  |  |  |  |  |
| The application process was easy to follow |  |  |  |  |  |
| **Structured modules** |  |  |  |  |  |
| There was a variety of modules |  |  |  |  |  |
| Duration of the modules was adequate |  |  |  |  |  |
| Assessments were fair |  |  |  |  |  |
| **Research project** |  |  |  |  |  |
| Guidelines were clear |  |  |  |  |  |
| Support from supervisor was adequate |  |  |  |  |  |
| **General** |  |  |  |  |  |
| Doing the MSc in Clinical Epidemiology influenced my career path |  |  |  |  |  |

- 1. What do you think worked well? _____________________________________________
  2. What do you think did not work well? ______________________________________________
  3. Do you have any suggestions how to improve the masters programme in clinical epidemiology? __________________________________________________________________________

1. What activities are you engaged in related to clinical epidemiology?
   1. Conducting research Yes / No

If yes,

- - - Number of publications ##
    - Please list the citations of your publications ______________________________
    - Number of conference presentations (oral or poster) ##
    - Number of ongoing research projects ##
    - Topics of ongoing research _______________________________
  1. Promoting use of evidence in healthcare decision making Yes / No

If yes, please describe the activities you are engaged in _______________________________________________________________________

- 1. Teaching clinical epidemiology Yes / No

If yes,

- Who do you teach? _________________________
- Where / at which institution(s)? _________________________
- What topics to you cover? ______________________________
- Which teaching methods are you using? _______________________

10. Any other comments? ___________________________________
